# Using a Chatbot as an Alternative Approach for In-Person Toothbrushing Training During the COVID-19 Pandemic: Comparative Study

**DOI:** 10.2196/39218

**Published:** 2022-10-21

**Authors:** Samerchit Pithpornchaiyakul, Supawadee Naorungroj, Kittiwara Pupong, Jaranya Hunsrisakhun

**Affiliations:** 1 Department of Preventive Dentistry Faculty of Dentistry Prince of Songkla University Hatyai, Songkhla Thailand; 2 Improvement of Oral Health Care Research Unit Faculty of Dentistry Prince of Songkla University Hatyai, Songkhla Thailand; 3 Department of Conservative Dentistry Faculty of Dentistry Prince of Songkla University Hatyai, Songkhla Thailand; 4 Maelan Hospital Maelan, Pattani Thailand

**Keywords:** mHealth, tele-dentistry, digital health, chatbot, conversional agents, oral hygiene, oral health behaviors, protection motivation theory, young children, caregiver, in-person toothbrushing training, COVID-19

## Abstract

**Background:**

It is recommended that caregivers receive oral health education and in-person training to improve toothbrushing for young children. To strengthen oral health education before COVID-19, the 21-Day FunDee chatbot with in-person toothbrushing training for caregivers was used. During the pandemic, practical experience was difficult to implement. Therefore, the 30-Day FunDee chatbot was created to extend the coverage of chatbots from 21 days to 30 days by incorporating more videos on toothbrushing demonstrations and dialogue. This was a secondary data comparison of 2 chatbots in similar rural areas of Pattani province: Maikan district (Study I) and Maelan district (Study II).

**Objective:**

This study aimed to evaluate the effectiveness and usability of 2 chatbots, 21-Day FunDee (Study I) and 30-Day FunDee (Study II), based on the protection motivation theory (PMT). This study explored the feasibility of using the 30-Day FunDee chatbot to increase toothbrushing behaviors for caregivers in oral hygiene care for children aged 6 months to 36 months without in-person training during the COVID-19 pandemic.

**Methods:**

A pre-post design was used in both studies. The effectiveness was evaluated among caregivers in terms of oral hygiene practices, knowledge, and oral health care perceptions based on PMT. In Study I, participants received in-person training and a 21-day chatbot course during October 2018 to February 2019. In Study II, participants received only daily chatbot programming for 30 days during December 2021 to February 2022. Data were gathered at baseline of each study and at 30 days and 60 days after the start of Study I and Study II, respectively. After completing their interventions, the chatbot's usability was assessed using open-ended questions. Study I evaluated the plaque score, whereas Study II included an in-depth interview. The 2 studies were compared to determine the feasibility of using the 30-Day FunDee chatbot as an alternative to in-person training.

**Results:**

There were 71 pairs of participants: 37 in Study I and 34 in Study II. Both chatbots significantly improved overall knowledge (Study I: *P*<.001; Study II: *P*=.001), overall oral health care perceptions based on PMT (Study I: *P*<.001; Study II: *P*<.001), and toothbrushing for children by caregivers (Study I: *P*=.02; Study II: *P*=.04). Only Study I had statistically significant differences in toothbrushing at least twice a day (*P*=.002) and perceived vulnerability (*P*=.003). The highest overall chatbot satisfaction was 9.2 (SD 0.9) in Study I and 8.6 (SD 1.2) in Study II. In Study I, plaque levels differed significantly (*P*<.001).

**Conclusions:**

This was the first study using a chatbot in oral health education. We established the effectiveness and usability of 2 chatbot programs for promoting oral hygiene care of young children by caregivers. The 30-Day FunDee chatbot showed the possibility of improving toothbrushing skills without requiring in-person training.

**Trial Registration:**

Thai Clinical Trials Registry TCTR20191223005; http://www.thaiclinicaltrials.org/show/TCTR20191223005 and TCTR20210927004; https://www.thaiclinicaltrials.org/show/TCTR20210927004

## Introduction

Early childhood caries (ECC) continues to be a significant public health problem worldwide, including in Thailand. ECC can have a negative impact on children's quality of life [1-3] and cost societies and families [4]. Caries is a multifactorial disease in which oral hygiene is a crucial risk factor for developing ECC [5-8]. For young children, having caregivers clean their teeth twice daily with fluoride toothpaste is recommended to prevent ECC [9]. In Thailand, ECC and oral hygiene care for children younger than 3 years remain unsatisfactory. The national oral health survey in 2017 reported a prevalence of dental caries in children aged 3 years of 52.9% and an average number of caries of 2.8 teeth per person [10]. The approach to improving caregivers' toothbrushing behavior with young children has emphasized oral health education in conjunction with in-person training for caregivers [11-14]. However, this personalized approach necessitates time and human resources.

In recent decades, chatbots have been introduced as a new way to improve person-centered health care, particularly to enhance the efficiency of delivery of primary health care services such as health education and counseling support [15,16], which has been shown to increase access to and the quality of services and health information while using fewer human resources [17,18]. Chatbots are computer programs that mimic human conversations using text or voice messages. The technology could be a set of rule-based algorithms or machine learning techniques such as natural language processing to automate some parts of the dialogue [19]. Chatbots are used in a wide range of health care settings [17]; however, they are rare in dentistry.

Zhang et al [20] proposed an artificial intelligence (AI) chatbot behavior change model as a theoretical framework for guiding the design and evaluation of chatbots. This model consists of the following 4 primary components: (1) defining the qualities of the chatbot and gaining an understanding of the user's context, (2) constructing relational ability, (3) building persuasive conversational capacity such as using behavioral change models, and (4) evaluating methods and outcomes. Among the theories of behavioral change for creating persuasive conversational capacity proposed by Zhang et al [20], the protection motivation theory (PMT) [21,22] has been widely accepted [23-25]. PMT is explained by the interplay between threat and coping appraisals, which results in protective health behavior [23]. Threat appraisal combines perceived severity (perceptions of the extent of harm) and perceived vulnerability (perceptions of the likelihood of experiencing harm). Coping appraisal comprises response efficacy (confidence in the effectiveness of the advice in reducing or preventing potential damage) and self-efficacy (belief in one's ability to carry out the recommended behavior successfully), minus response costs (the perceived or actualized costs associated with the practice of the recommended behavior) [21,22]. PMT has been demonstrated to be applicable for behavior change and to have a positive effect on adaptive intentions or behaviors in the varied communication approaches [11,24-26] for which the theory has been suggested for individual and community interventions. Numerous oral health studies have applied and assessed protection motivation variables to determine behavioral intention to self-protective action [11,27], but none have utilized chatbots as an intervention for protective behavior change in dentistry. Therefore, this study intended to use the AI chatbot behavior change model as a framework to enhance the chatbot's usability, satisfaction, and design outcome measures, while PMT was specifically applied to design the content and ways to modify toothbrushing behavior.

A chatbot-based mobile application, a novel PMT-based solution to prevent ECC in Thailand, was launched through the WowBot project in 2018 before the COVID-19 epidemic. The “21-Day FunDee” chatbot and in-person toothbrushing training were implemented in 6 study centers, 5 in rural areas and 1 in an urban area. However, during COVID-19, caregivers with children had difficulty accessing hospitals and other health care service providers, making in-person training impossible. As a result, a modified version entitled “30-Day FunDee” was created to overcome these obstacles, reduce the burden of oral health promotion on health care workers, and decrease the risk of COVID-19 exposure. More video demonstrations and communication focused on in-person practice of toothbrushing techniques and increased motivation for caregivers to improve oral health care for their young children were included in this version. Both chatbots have already been evaluated in sociodemographically comparable populations over different time periods.

Therefore, this secondary data analysis aimed to evaluate the effectiveness and usability of the chatbots before and during the COVID-19 pandemic. In 2 study settings, we demonstrated what we learned about the application of chatbots to promote caregivers’ oral health care practices for young children and the feasibility of using chatbots to improve toothbrushing abilities without actual practice.

## Methods

### Study Location and Phases

This study performed secondary data analysis of data from the WowBot project, which was conducted between 2018 and 2024. We chose 2 study settings in Pattani province (Study I in Maikan district and Study II in Maelan district) with comparable socioeconomic backgrounds to illustrate how innovative chatbots encourage caregivers to provide oral health care for their young children in 2 situations: before and during the COVID-19 pandemic.

The WowBot project consisted of 3 phases. Phase I included a scoping review and the development of a chatbot based on PMT. Phase II comprised an assessment of the usability and effectiveness of the first chatbot called “21-Day FunDee” together with in-person toothbrushing training in 6 study centers (Pattani, Phangha, Trang, Songkhla, Nakhonsrithammarat, and Patthalung provinces). Toward the end of phase II, during the COVID-19 pandemic, the chatbot “21-Day FunDee” content was modified with the addition of the following video clips: toothbrushing technique, an examination of dental plaque, and child behavioral management during toothbrushing. The effectiveness of “30-Day FunDee” was evaluated in another community (Maelan District, Pattani province) during the lockdown measures in Thailand. Phase III, an ongoing study, aims to improve chatbot performance and conduct a long-term evaluation of clinical outcomes.

### Ethics Approval

The 2 studies were registered with the Thai Clinical Trials Registry and approved by the Faculty of Dentistry, Prince of Songkla University Institutional Review Board (EC6208-031 and EC6407-053).

### Chatbot Characteristics and Development

**Figure 1:**
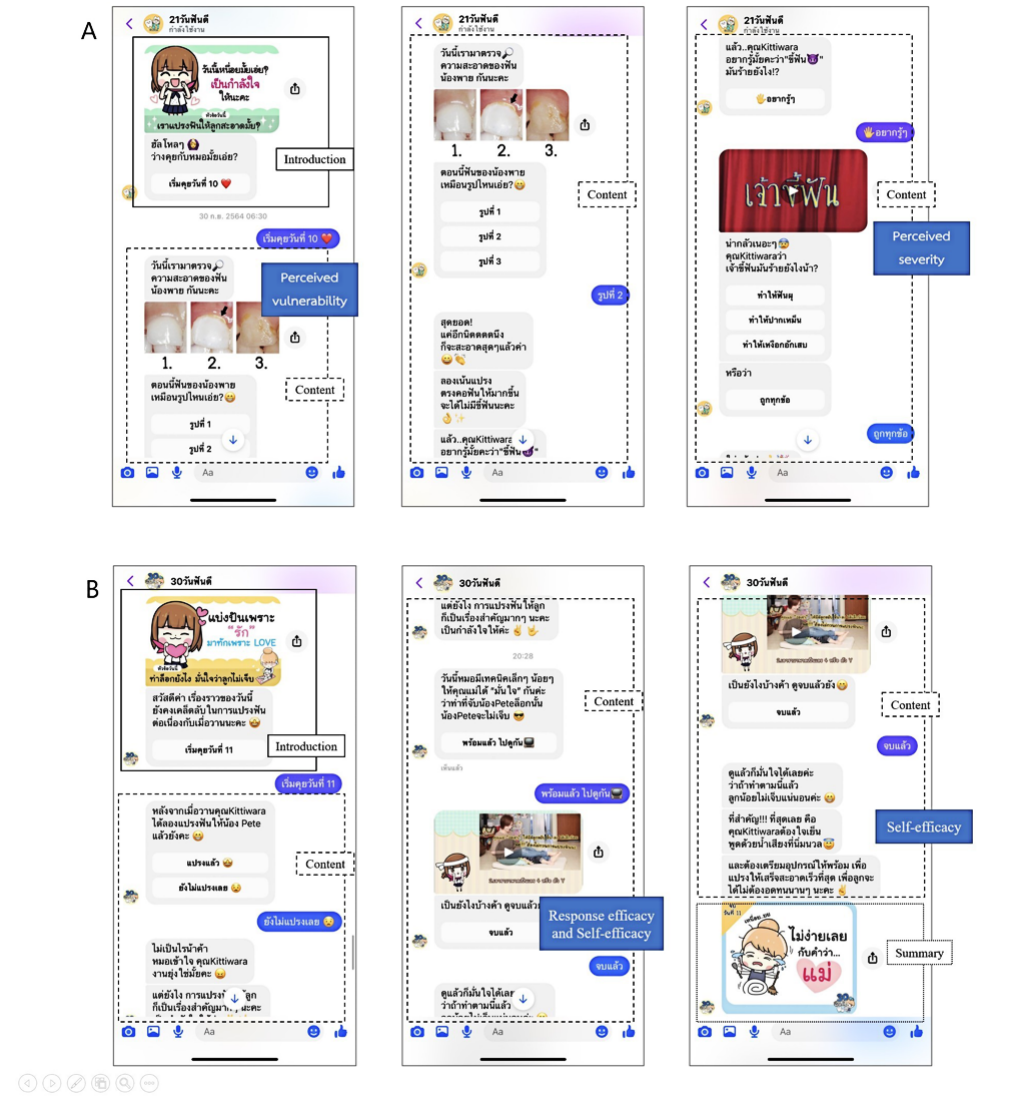
Examples of user interfaces of the Facebook Messenger–based (A) 21-Day FunDee and (B) 30-Day FunDee including 3 essential elements: greeting; content; and summary containing games, infographics, videos, and friendly conversations.

**Figure 2:**
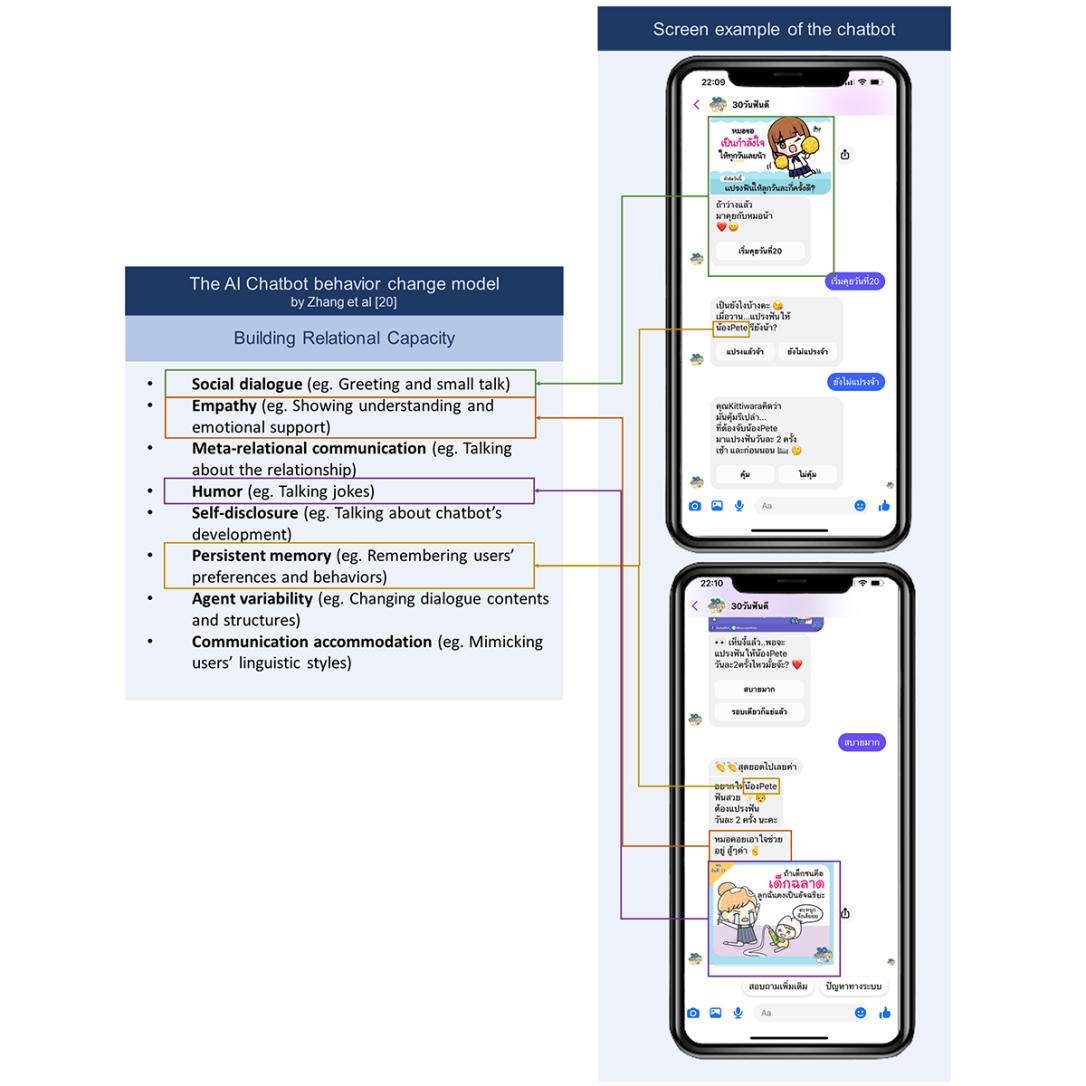
Screenshots showing examples of using the artificial intelligence (AI) chatbot behavior change model.

Regarding the design of the chatbots, we applied all 4 components from the model by Zhang et al [20]. For example, in component 1, when designing chatbot characteristics and understanding users’ backgrounds, we designed a young female doctor with a friendly, cheerful, and compassionate personality. For component 2, building relational capacity, our chatbot sent caregivers daily funny greeting cards, discussed their challenges, offered emotional support, and concluded with an image or infographic containing an inspirational quote. For component 3, building persuasive conversational capacity, we delivered daily content containing (1) explicit, understandable oral health care knowledge and (2) PMT-based oral health behavior improvement. Last, we applied the fourth component, which was to evaluate mechanisms and outcomes by asking open-ended questions about satisfaction, usage patterns, and how people brushed their teeth (Figures 1 and 2).

The rule-based strategy was used to construct the chatbot flow of “21-Day FunDee” and its modified version, “30-Day FunDee,” and the chatbots were developed utilizing the Chatfuel platform. PMT constructs, including perceived severity, perceived vulnerability, response efficacy, and self-efficacy, were used to guide the development of the chatbot content. The chatbots operate on Facebook messenger over the course of 21 and 30 daily sessions, respectively. Through various interactive conversation flows, the chatbots were designed to engage and motivate the users with interesting conversation with rule-based agents. Each session was to run around 3 minutes to 5 minutes and comprises text, video clips, or infographics. The first developed chatbot application, “21-Day FunDee,” was reviewed by 2 dentists specializing in community health, while the modified version, “30-Day FunDee,” was reviewed by 2 pedodontists. Experts determined the validity of the content based on its accuracy, relevance, and conversational flow. We recruited 10 volunteers to test each chatbot in the following aspects: conversational flow, onboarding, understanding, navigation, response time, and the chatbot’s personality.

### Setting and Participants

A quasi-experimental design (pretest and posttest) was used to evaluate the chatbots’ effectiveness and usability. Community health-promoting databases were used to identify potential subjects for each study setting. Potential participants in Study I were caregivers and children aged 6 months to 36 months who went to a health-promoting hospital for a vaccination or a health checkup. Potential participants for Study II were caregivers and children aged 6 months to 36 months, but they were contacted and provided with information regarding the study protocol via telephone. Participants for Study I were recruited from October 2018 to February 2019, with a total of 37 pairs agreeing to participate in the study. From December 2021 to February 2022, 34 pairs of participants were recruited for Study II.

Prior to data collection, participants were asked to provide written informed consent (Study I) or verbal consent (Study II). Throughout the study period, caregivers were required to have a smartphone connected to the internet and have or agree to apply for Facebook messenger. The child was required to have at least one tooth and have no serious medical problems affecting oral health status (eg, Down syndrome). Parent-child pairs were excluded from the study if they could not communicate in the Thai language or refused to consent to the study.

### Interventions

After consenting to the study, the caregivers were trained to use the chatbots via their mobile devices. For Study I, caregivers were also given in-person instructions on toothbrushing technique, toothbrush selection, amount of toothpaste, and children’s positioning while brushing teeth. The trainers used a doll to demonstrate toothbrushing (scrub technique); then, the caregiver practiced toothbrushing with their child. The training session lasted 10 minutes to 15 minutes. As previously mentioned, due to the COVID-19 pandemic, in-person toothbrushing training was replaced with a video clip in Study II. The chatbot administrator observed the user engagement during the trial and assisted with resolving technical problems.

### Data Collection and Outcome Assessment

There were 2 primary outcomes in this study: the effectiveness and usability of the chatbots. The chatbots’ effectiveness was assessed in terms of changes in knowledge, oral health care perceptions based on PMT, and practices in oral hygiene care of young children. Both studies used a structured questionnaire designed to gather information on sociodemographic characteristics and oral health knowledge, perceptions, and practices at baseline and follow-up. Oral health knowledge questions comprised 11 items covering the following: appropriate time to begin brushing, frequency of brushing, brushing method, fluoride toothpaste, and child behavior management. A correct answer received 1 point, while an incorrect answer received 0 points. Questions about perceptions collected information about perceived severity, perceived vulnerability, response efficacy, and self-efficacy. The answer options were “positive perception” (3 points), “uncertain” (2 points), and “negative perception” (1 point). Oral hygiene practices were assessed using 4 categorical questions: brushing by a caregiver, frequency of toothbrushing per day, fluoride toothpaste usage, and the amount of toothpaste used.

The content validity and construct validity of the questionnaire were assessed by 2 experts for Study I and 3 experts for Study II. In both studies, face validity was determined through questionnaire-based pilot testing with 15 participants. A face-to-face interview by trained interviewers was used to collect data for Study I, while a self-administered online questionnaire via a Google Form was applied during the COVID-19 pandemic for Study II. In addition to the questionnaire, 1 dentist examined oral hygiene status under natural light using a disposable plastic straw and mouth mirror at baseline and follow-up in Study I. The amount of visible plaque found on the buccal surface of all erupted teeth was scored (0=no visible plaque; 1=presence of plaque).

**Figure 3:**
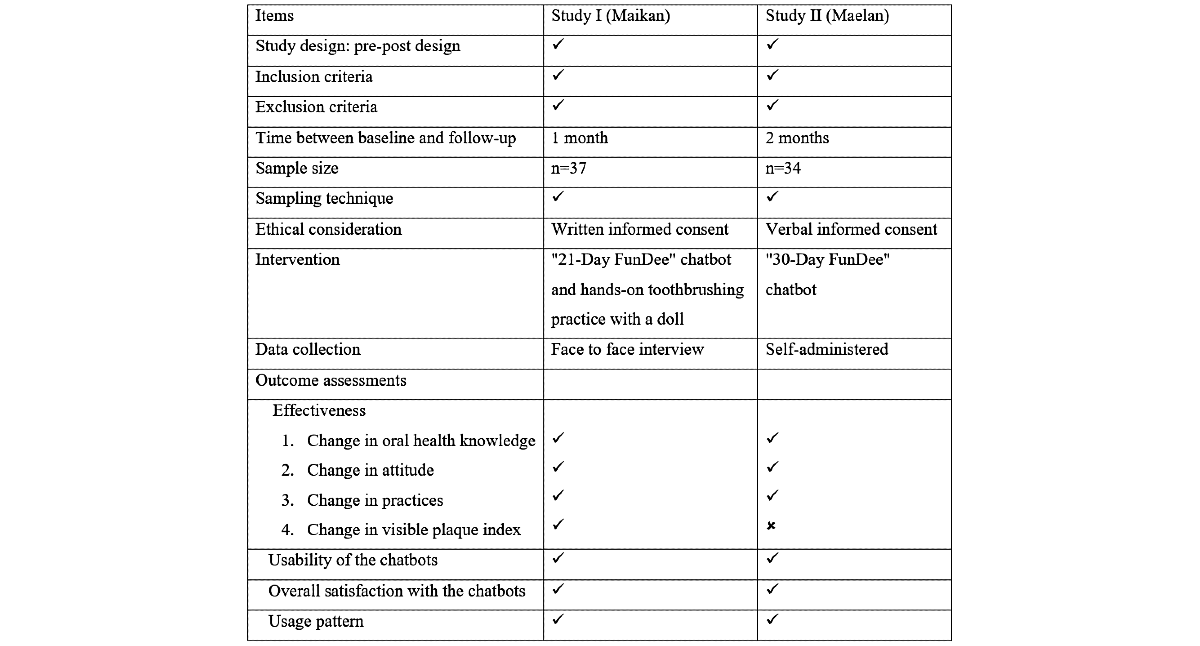
Comparison of the methodologies used in the 2 study settings.

Caregivers were asked to provide feedback on satisfaction with the chatbot at the last session (21st or 30th day). Open-ended questions were used to collect caregivers’ suggestions. The overall satisfaction score rating ranged from 0 to 10 (0=very dissatisfied; 10=very satisfied). For Study II, an additional survey was administered using an in-depth interview (n=8). The usage patterns and days of engagement with the chatbots were recorded in log files (Figure 3).

### Data Analysis

The data were statistically analyzed using the free, open-access statistical software PSPP version 1.2.0 for Windows. Cronbach *α* was used to determine the questionnaire’s internal consistency on overall oral health care perceptions based on PMT (*α*=.81 for Study I, *α*=.83 for Study II). Descriptive statistics were used to describe the baseline characteristics of study participants and responses to the questionnaires. The scores for perception and knowledge questions were normalized to 1 by summarizing the response scores of each question and dividing by the number of questions and the highest score. Then, means and standard deviation for each item and the overall score of the knowledge and perception questionnaires were calculated. The McNemar test was used to determine if there was a significant change in the proportions (before and after) of oral hygiene practices, and the Student paired *t* test was used to determine whether there was a statistically significant change in the mean scores for knowledge, perception, and plaque levels. The percentage of caregivers who finished the chatbot session and the average number of days using chatbots were calculated.

## Results

The sample comprised 71 pairs of participants: 37 for Study I and 34 for Study II. Similar sociodemographic characteristics were observed in the 2 studies (Table 1).

Both studies revealed a significant increase in toothbrushing for children by caregivers. Additionally, an increase in toothbrushing at least twice daily by caregivers for children was reported, but only Study I showed a significant difference (*P*=.02), with a 22% increase. The percentage of caregivers who used fluoride toothpaste and the amount of toothpaste used did not differ significantly. The plaque level significantly reduced after the intervention in Study I (Table 2).

Both the 21-day and 30-day chatbot applications significantly improved overall oral health care perceptions based on PMT, and every category, except perceived vulnerability, in Study II was not statistically different (Table 3).

Both chatbot applications statistically significantly increased overall knowledge and specific knowledge, such as brushing techniques and use of fluoride toothpaste. Additionally, the chatbot in Study I demonstrated a significantly greater understanding of the appropriate time to start toothbrushing and how to manage child behavior while brushing (Table 4).

Table 5 summarizes chatbot engagement and satisfaction with both chatbots. Satisfaction scores for Studies I and II were very high, at 9.2 and 8.6 out of 10, respectively. Almost all participants expressed that their respective chatbot was enjoyable, content-rich, capable of empathetic interaction, and worthy of assisting them improve their behaviors. Additionally, the users were impressed by the multimedia elements such as video and infographics for compelling storytelling and artwork. Quite a few participants raised concerns about the platform’s stability. Some participants in Study I mentioned that in-person toothbrushing training made them more confident at toothbrushing and eager to clean their children’s teeth.

**Table 1:** Characteristics of the samples in Studies I and II.

Characteristics			Study I (37 child-parent pairs)		Study II (34 child-parent pairs)
Child age (months), mean (SD)			16.4 (6.1)		20.1 (8.0)
**Primary caregiver, n (%)**					
	Mother	35 (95)		30 (88)	
	Other	2 (6)		4 (12)	
Caregiver age (years), mean (SD)			31.9 (9.0)		32.3 (10.1)
**Caregiver education, n (%)**					
	Primary school	3 (8)		6 (18)	
	Junior high school	4 (11)		6 (18)	
	High school	16 (43)		10 (29)	
	Diploma or more	14 (38)		12 (35)	
**Caregiver occupation, n (%)**					
	Housewife	5 (13.5)		16 (47.1)	
	Employee	23 (62.2)		8 (23.5)	
	Agriculture	0 (0)		2 (5.9)	
	Business owner	3 (8.1)		3 (8.8)	
	Government	6 (16.2)		5 (14.7)	
**Religion, n (%)**					
	Buddhist	1 (3)		4 (12)	
	Muslim	36 (97)		30 (88)	
Number of children in their house/siblings, mean (SD)			2.0 (1.1)		2.3 (1.5)

**Table 2:** The effects of the chatbot application on oral health care practices for young children in Studies I and II.

Oral health practices		Study I					Study II			
		Before, n (%)	After, n (%)	Chi-square *(df)*	*P* value	Before, n (%)		After, n (%)	Chi-square *(df)*	*P* value
**Toothbrushing for children by caregiver**										
	Yes	28 (76)	36 (97)	6.40 (1)	.02^a^	22 (65)		30 (88)	5.33 (1)	.04^a^
	No	9 (24)	1 (3)			12 (35)		4 (12)		
**Frequency of brushing by caregiver**										
	Not everyday	13 (46)	3 (8)	12.23 (2)	.002	0 (0)		0 (0)	N/A^b^	.69^c^
	Once a day	5 (18)	12 (33)			3 (14)		3 (10)		
	Twice or more a day	10 (36)	21 (58)			19 (86)		27 (90)		
Fluoride toothpaste usage		22 (96)	36 (100)	N/A	.39^c^	13 (87)		25 (100)	N/A	.14^c^
**Amount of toothpaste**										
	Smear	15 (65)	22 (61)	0.10 (1)	.75	16 (76)		25 (93)	N/A	.22^c^
	Pea or regular size	8 (35)	14 (39)			5 (24)		2 (7)		
Plaque score^d^		0.48 (0.33)	0.18 (0.21)	6.82 (36)	<.001	N/A		N/A	N/A	N/A

^a^McNemar-test.

^b^N/A: not applicable.

^c^Fisher exact test.

^d^Mean (SD).

**Table 3:** The effects of the chatbot application on oral health care perceptions based on protection motivation theory in Studies I and II.

Perceptions^a^	Study I					Study II			
	Pretest, mean (SD)	Posttest, mean (SD)	*t* test *(df)*^b^	*P* value	Pretest, mean (SD)		Posttest, mean (SD)	*t* test *(df)*^b^	*P* value
Perceived severity	0.47 (0.33)	0.79 (0.26)	4.94 (36)	<.001	0.73 (0.20)		0.86 (0.15)	4.03 (33)	<.001
Perceived vulnerability	0.46 (0.51)	0.78 (0.42)	3.15 (36)	.003	0.83 (0.24)		0.89 (0.19)	1.07 (33)	.29
Response efficacy	0.57 (0.24)	0.90 (0.18)	7.76 (36)	<.001	0.68 (0.14)		0.79 (0.16)	3.89 (33)	<.001
Self-efficacy	0.71 (0.24)	0.89 (0.18)	4.15 (36)	<.001	0.72 (0.15)		0.80 (0.15)	2.64 (33)	.01
Overall perceptions	0.58 (0.19)	0.86 (0.16)	7.67 (36)	<.001	0.74 (0.12)		0.83 (0.12)	4.36 (33)	<.001

^a^The scores were normalized to 1.

^b^Paired *t* tests were used to compare the difference between pretest and posttest scores.

**Table 4:** The effects of the chatbot application on knowledge in Studies I and II.

Knowledge^a^	Study I					Study II			
	Pretest, mean (SD)	Posttest, mean (SD)	*t* test *(df)*^b^	*P* value	Pretest, mean (SD)		Posttest,mean (SD)	*t* test *(df)*^b^	*P* value
Appropriate time to start toothbrushing	0.22 (0.42)	0.86 (0.35)	7.33 (36)	.001	0.38 (0.49)		0.59 (0.50)	1.87 (33)	.07
Frequency of toothbrushing	0.78 (0.42)	0.80 (0.34)	1.00 (36)	.32	0.59 (0.31)		0.59 (0.34)	0.00 (33)	.99
Brushing method	0.80 (0.33)	1.00 (0)	3.83 (36)	.001	0.64 (0.17)		0.75 (0.17)	3.40 (33)	.002
Fluoride toothpaste	0.82 (0.27)	1.00 (0)	3.97 (36)	<.001	0.38 (0.49)		0.62 (0.49)	2.27 (33)	.03
Child behavior management	0.81 (0.29)	0.96 (0.14)	2.93 (36)	.006	0.69 (0.25)		0.72 (0.25)	1.00 (33)	.33
Overall knowledge	0.73 (0.21)	0.94 (0.09)	6.32 (36)	<.001	0.53 (0.26)		0.66 (0.23)	3.50 (33)	.001

^a^The scores were normalized to 1.

^b^Paired *t* tests were used to compare the difference between pretest and posttest scores.

**Table 5:** Engagement and satisfaction with the chatbot in Studies I and II.

Engagement and satisfaction			Study I		Study II
**Chatbot engagement**					
	Full program engagement, n (%)	30 (81)		25 (74)	
	Days of engagement, mean (SD)	19.9 (4.9)		24.2 (2.8)	
Days of engagement per week, mean (SD)			6.4 (1.5)		5.7 (1.7)
Satisfaction with the bot (0-10), mean (SD)			9.2 (0.9)		8.6 (1.2)

## Discussion

To the authors' knowledge, this is the first study to use a chatbot-mediated intervention for oral health both prior to and during the COVID-10 pandemic. This study presents the effectiveness of both the 21-day FunDee chatbot with in-person training and the 30-day FunDee chatbot application in improving the child oral hygiene care by caregivers. Additionally, it demonstrated the potential for chatbots to serve as an alternative for in-person training for skills such as toothbrushing.

Both studies resulted in a significant improvement in toothbrushing by caregivers for children, with greater percentages reported than by other studies conducted in similar age groups utilizing traditional oral health education with or without in-person toothbrushing training [12]. Toothbrushing for children by caregivers has been shown as a key factor influencing the quality of plaque reduction for these young children compared with child self-toothbrushing [9].

In Study I, the percentage of caregivers who brushed their children's teeth twice daily increased from 36% to 58%, a significant increase of approximately 22%. In contrast, few changes were observed in Study II, possibly as a result of better baseline toothbrushing practices. In both studies, all caregivers eventually used fluoride toothpaste, which may result in long-term benefits for caries control [28]. Brushing teeth twice a day moderately increased, similar to a study with toddlers aged 9 months to 18 months in Thailand utilizing PMT with in-person toothbrushing training, which reported an increase from 11% to 42% over 1 year, compared with an increase from 11% to 16% in the control group receiving routine care [11]. This was consistent with a participatory approach intervention for caregivers of children younger than 6 years that included 90-minute, small-group sessions providing educational information, direct instruction, practice, and peer-to-peer problem solving. After 4 weeks to 8 weeks of intervention, improvement ranged from 59% to 89% [29]. It is worth noting that all other studies used human resources to accomplish the changes. In a meta-analysis, infrequent brushing had a significant impact on the incidence and increment of carious lesions in deciduous teeth (odds ratio 1.75, 95% CI 1.49-2.06) [30].

Study I demonstrated remarkable plaque reduction (62.5%) at the 1-month evaluation, which was similar to a study using a gamification application for mothers to reduce plaque accumulation in children aged 4 years to 5 years (plaque reduction of 50%) [31]. Both studies demonstrated a significant increase in oral health care knowledge, particularly regarding toothbrushing technique and the use of fluoride toothpaste. However, the caregivers believed that once per day was sufficient for brushing their children's teeth. It is necessary to gain a deeper understanding of caregivers' motivations and beliefs to improve chatbot conversations. Study I demonstrated slightly more progress in overall oral health care knowledge than Study II, which may be explained by the lower beginning knowledge score and more extended evaluation period in Study II.

PMT was used to develop the 2 chatbots aiming to improve toothbrushing behavior and engagement. Our 2 studies showed a high success rate in improving overall caregivers' perceptions except for perceived vulnerability, observed in Study I only. It is possible that, in Study I, in-person training raised the participants’ awareness by showing them their children’s level of plaque, while 30-Day FunDee persuaded caregivers to check their children's plaque and compare it with infographics provided by chatbot. To increase the motivation to change, Study II may have enhanced perceived vulnerability using AI technology to compare plaque levels based on photographs of each participant that were more contextually relevant.

In-person training is a powerful way to increase one’s ability and empower confidence in toothbrushing [13,14]. A study demonstrated that increased perceived severity and self-efficacy via in-person training for toothbrushing techniques can reduce plaque levels on a long-term basis [11]. Additionally, Finlayson et al [32] found that increased maternal oral health–related self-efficacy is associated with increased frequency of toothbrushing in children aged 1 year to 5 years. Self-efficacy is a crucial aspect of PMT and has been identified as a factor that enables individuals to adhere to healthier behaviors and predicts a range of health behaviors including oral self-care [33-35]. In our study, we discovered that the chatbot, 30-Day FunDee, effectively improves toothbrushing practices, self-efficacy, response efficacy, and threat perceptions in terms of perceived severity. Thus, this chatbot might be used as an alternative for in-person training for toothbrushing for young children by caregivers.

Interestingly, both studies had high engagement rates (6.4 days and 5.7 days per week in Study I and Study II, respectively) compared with other studies on chatbots in health, such as the study by Jang et al [36] that reported 5.1 days per week of chatbot engagement with a 4-week chatbot course and the study by Fitzpatrick et al [37] that reported 6.1 days per week of chatbot adherence with a 14-day chatbot course. It is noticeable that a longer period for chatbot delivery is related to a decreased engagement rate. Engagement with the chatbot in our study may have resulted from the high satisfaction score for the chatbots. The chatbots were satisfying since they contain attractive multimedia; understandable content; friendly, empathetic dialogue; and utility as well as being easy to use. This is consistent with other studies showing high acceptability via chat enjoyment, bonding, creation of social and emotional relationships, ease of use, usefulness, and a desire to use [38,39].

Based on our experience, the model by Zhang et al [20] is useful for planning the overall conversational flow and creating more humanized chatbots. Incorporating content and behavioral change theory into the conversational flow was the most challenging aspect of achieving harmony. The conversational flow reflected, to some extent, what we had used to successfully motivate patients to improve their oral health behavior and what we had learned by applying theory to practice.

This secondary data analysis has some limitations. First, both studies used a pre-post design that may have a maturity bias; therefore, the chatbot’s effectiveness in improving oral health behavior may be overestimated. Second, although our study was conducted with similar research methodology, the interview procedure and follow-up period differed. Study I used face-to-face interviews and a shorter follow-up period of 9 days after the intervention ended; therefore, the study may be subject to examiner bias, and the results may reflect the chatbot’s short-term effect. Although Study II used a self-administered online questionnaire, its validity could be compromised if individuals responded unintentionally, and a longer follow-up time could influence memory retention. To generalize the results of this study, randomized trials in different groups and a longer-term evaluation of caries prevention should be conducted in future studies. Furthermore, to improve the effectiveness of chatbots, adaptive learning and AI-based conversation should be incorporated.

This study introduced chatbot applications as a new normal approach to oral health education. We demonstrated the effectiveness of using chatbots to empower caregivers of young children to perform oral hygiene care for the child prior to and during the COVID-19 pandemic and showed the possibility of using chatbots to improve toothbrushing abilities without actual in-person training.

## Abbreviations

AI: artificial intelligence
ECC: early childhood caries
PMT: protection motivation theory
